# Comparative genome analysis of *Weissella ceti*, an emerging pathogen of farm-raised rainbow trout

**DOI:** 10.1186/s12864-015-2324-4

**Published:** 2015-12-22

**Authors:** Henrique C. P. Figueiredo, Siomar C. Soares, Felipe L. Pereira, Fernanda A. Dorella, Alex F. Carvalho, Júnia P. Teixeira, Vasco A. C. Azevedo, Carlos A. G. Leal

**Affiliations:** AQUACEN, National Reference Laboratory for Aquatic Animal Diseases, Ministry of Fisheries and Aquaculture, Federal University of Minas Gerais, Belo Horizonte, MG Brazil; Laboratory of Cellular and Molecular Genetics, Institute for Biological Science, Federal University of Minas Gerais, Belo Horizonte, MG Brazil; Veterinary School, Department of Preventive Veterinary Medicine, Federal University of Minas Gerais, Av. Antônio Carlos 6627, Pampulha, Belo Horizonte, 30161-970 MG Brazil

**Keywords:** *Weissella ceti*, Tilapia, Adhesins, Antibiotic resistance, Pathogenicity islands, wgMLST, Hemolysin, Cold adaptation

## Abstract

**Background:**

The genus *Weissella* belongs to the lactic acid bacteria and includes 18 currently identified species, predominantly isolated from fermented food but rarely from cases of bacteremia in animals. Recently, a new species, designated *Weissella ceti*, has been correlated with hemorrhagic illness in farm-raised rainbow trout in China, Brazil, and the USA, with high transmission and mortality rates during outbreaks. Although *W. ceti* is an important emerging veterinary pathogen, little is known about its genomic features or virulence mechanisms. To better understand these and to characterize the species, we have previously sequenced the genomes of *W. ceti* strains WS08, WS74, and WS105, isolated from different rainbow trout farms in Brazil and displaying different pulsed-field gel electrophoresis patterns. Here, we present a comparative analysis of the three previously sequenced genomes of *W. ceti* strains from Brazil along with *W. ceti* NC36 from the USA and those of other *Weissella* species.

**Results:**

Phylogenomic and orthology-based analyses both showed a high-similarity in the genetic structure of these *W. ceti* strains. This structure is corroborated by the highly syntenic order of their genes and the neutral evolution inferred from Tajima’s D. A whole-genome multilocus sequence typing analysis distinguished strains WS08 and NC36 from strains WS74 and WS105. We predicted 10 putative genomic islands (GEI), among which PAIs 3a and 3b are phage sequences that occur only in WS105 and WS74, respectively, whereas PAI 1 is species specific.

**Conclusions:**

We identified several genes putatively involved in the basic processes of bacterial physiology and pathogenesis, including survival in aquatic environment, adherence in the host, spread inside the host, resistance to immune-system-mediated stresses, and antibiotic resistance. These data provide new insights in the molecular epidemiology and host adaptation for this emerging pathogen in aquaculture.

**Electronic supplementary material:**

The online version of this article (doi:10.1186/s12864-015-2324-4) contains supplementary material, which is available to authorized users.

## Background

The genus *Weissella* is a recently classified taxonomic group within the lactic acid bacteria (LAB), closely related to the genera *Leuconostoc* and *Oenococcus* [1, 2]. The genus *Weissella* was established in 1993 and to date, 19 names have been attributed to 18 species (*W. cibaria* is considered a synonym of *W. kimchii*) [3]. The majority of *Weissella* strains have been isolated from vegetables, fermentative substrates, meat, meat products, and the gastrointestinal tracts of some animal species, insects, and humans [4–6]. Several species, including *W. confusa* and *W. cibaria*, have been associated with rare cases of bacteremia in humans and animals [7–10].

Although the majority of *Weissella* strains, like the many other LAB, are considered nonpathogenic to animals, recent outbreaks of hemorrhagic disease associated with a *Weissella* species with high mortality rates, have been described in farm-raised rainbow trout (*Oncorhynchus mykiss*), first in China [10] and then in Brazil [11] and the USA [12]. In 2011, during a study of the microbiota of beaked whales, Vela et al. isolated a Gram-positive rod-shaped bacterium from the brain, kidney, lymph nodes, and spleen of this mammal [13]. These isolates were ascribed to a new *Weissella* species, designated *Weissella ceti*. Analysis of 16S rRNA genes of the rainbow trout strains isolated in China, Brazil, and the USA showed that they belonged to the same species, and this emerging disease was called “weissellosis” [14, 15].

The ability of this pathogen to infect different target organs in fish (brain, spleen, liver, kidney, and intestine), its high transmission rate through water, and its contemporaneous occurrence on different continents suggest that lineages of *W. ceti*, unlike the other species of the genus *Weissella*, have adapted to a pathogenic lifestyle. The disease has been associated with water temperatures of ~15 °C in ponds in the outbreaks described in all countries, a temperature that inhibits the growth of *W. ceti* isolated from the beaked whale, supporting the suggestion that the strains have adapted to fish hosts [11, 13, 15].

The genetic traits and diversity of *W. ceti* are poorly understood. Welch and Good [12] described a high degree of similarity in the 16S rRNA gene sequences of strains isolated in China, Brazil, and the USA. Costa et al. [14] compared 34 strains isolated from eight different farms in Brazil using pulsed-field gel electrophoresis (PFGE) and showed that the strains belonged to a single PFGE type, divided into three clonally related PFGE patterns. At present, the genomes of four *W. ceti* strains have been sequenced, but with no further comparative genomic characterization [15–17].

Here, we present a comparative genomic analysis of these four *W. ceti* genomes and their relationships to other species of the genus *Weissella*. Our results provide new insight into the evolution, pathogenicity, and host adaptation of *W. ceti*.

## Methods

### Growth of *W. ceti* strains at 15 °C

The ability of *W. ceti* strains WS08, WS74, and WS105 to grow and survive in brain–heart infusion broth (BHI) for 15 days was evaluated. BHI was inoculated with bacterial cells of each strain, previously grown on sheep blood agar at 28 °C, and then incubated at 15 °C in an aerobic environment for 15 days. After the broth became turbid (positive growth), bacterial viability was checked daily by streaking a 10 μL aliquot onto 5 % sheep blood agar, which was then incubated at 28 °C for 48 h. The colonies were identified as described previously [11].

### Genome sequencing and assembly

The WS08 strain was sequenced and assembled as described in a previous work [16]. Two sequencing technologies were used: 200 bp fragment kit and long mate-pair kit, with an average insert size of 6000 bp, both on Ion Torrent Personal Genome Machine – PGM (Life Technologies, USA), described on Additional file 1. The assembly of the fragment library resulted in ten contigs (Additional file 2), using Mira Assembler version 3.9.18 [18], with parameters “genome,denovo,accurate -AS:urd = yes -AS:klrs = yes IONTOR_SETTINGS -AS:mrpc = 100”. A super scaffold of these contigs was generated by mapping the paired reads to contigs flanking regions using CLC Workbench 7.0 (Qiagen, USA), followed by coverage analysis. This processes consisted of testing all pairwise combinations of contigs, assuming a correct match when 20 % or more of the mapped read pairs anchored both contigs. Afterwards, the gaps were filled by performing successive recursive mappings of reads to gap regions of the scaffold, until overlapping regions were found. Finally, the circular genome, comprised of 1,355,853 bp, was checked with an in-house PFGE database [14] on BioNumerics version 6.6 (Applied Math, USA). The final sizes of the genomes corroborate the PFGE results, which showed an approximate genome size ranging from 1.40 to 1.49 Mb (Additional file 3). WS74 and WS105 were sequenced and assembled as described by Figueiredo et. al. [15]. In summary, the sequencing was made with a 200 bp fragment library kit in PGM system for both strains. Assemblies were performed with Newbler software (Roche, USA) version 2.9, with default parameters, and resulted in 19 and 20 contigs for WS74 and WS105, respectively. CONTIGuator 2.0 [19], with default parameters, was used to create a super scaffold for each strain, using WS08 as a reference genome. The gaps over rRNA operon regions were closed by extracting consensus sequences from the mapping of raw data over WS08 reference. The 13 and 14 remaining gaps of WS74 and WS105, respectively, were closed as described for WS08. WS74 and WS105 genomes were comprised of circular genomes with 1,389,513 and 1,390,396 bp, respectively.

### Genome annotation

For this work, the annotations of *W. ceti* WS08, WS74 and WS105 were updated in Prokka version 1.10, with default parameters, changing to perform BlastP similarity searches in nested databases, on this order: TrEMBL Uniprot containing only *Weissella* spp. proteins, RefSeq database containing only *Weissella* spp. proteins, and complete RefSeq database. After this automatic annotation, a manual curation of putative pseudogenes was performed using the software Artemis [20].

### Percentage similarity between all the sequenced species in the genus *Weissella*

A comparative genomic analysis was undertaken with the Gegenees software [21] to compare the percentage similarity between all the species of the genus *Weissella* whose genomes have been sequenced: *W. ceti* strains NC36, WS08, WS74, and WS105; *W. cibaria* KACC 11862, ff3PR, MG1, and AB3b; *W. confusa* LBAE C39-2; *W. halotolerans* DSM 20190; *W. koreensis* strains KACC 15510 and KCTC 3621; *W. paramesenteroides* ATCC 33313; *W. hellenica* Wikim14; *W. thailandensis* fsh4-2; and *W. oryzae* SG25 (Table 1). The resulting similarity matrix was used to generate a heatplot that was converted to the “.nexus” format for phylogenomic analysis. In this study, we used a sequence fragmentation length of 500 nucleotides and a threshold of 40 %.

**Table 1:** General features of *Weissella* species

Species	Strain	Country of isolation	Year of isolation	Farm^a^	GenBank Accession Number	Genome size (bp)	CDSs	Pseudogenes	rRNAs	tRNAs	tmRNAs	Hypothetical proteins (%)	Gene Mean length (bp)	Gene density (genes/kb)	Coding percentage	GC Content of Genes (%)	GC Content of Genome (%)
*Weissella ceti*	WS08	Brazil	2008	1	CP007588	1,355,853	1270	1	19	75	1	21.55	921	0.936	86.3	41.44	40.78
*Weissella ceti*	WS74	Brazil	2010	5	CP009223	1,389,513	1338	3	19	77	1	24.58	895	0.962	86.2	41.39	40.75
*Weissella ceti*	WS105	Brazil	2012	8	CP009224	1,390,396	1338	2	18	71	1	24.73	896	0.962	86.2	41.40	40.75
*Weissella ceti*	NC36^b^	USA	2011	–	ANCA00000000	1,352,640	1258	–	16	68	1^c^	23.29	932	0.930	86.6	41.46	40.76
*Weissella koreensis*	KACC 15510	–	–	–	CP002899	1,422,478	1335	–	15	56	1^c^	22.90	906	0.938	85.0	36.49	35.48
*Weissella koreensis*	KCTC 3621^b^	–	–	–	AKGG00000000	1,728,940	1672	–	17	61	1^c^	42.34	874	0.967	84.5	36.47	35.51
*Weissella cibaria*	KACC 11862^b^	–	–	–	AEKT00000000	2,317,857	2095	–	6	69	1^c^	26.54	962	0.903	87.0	46.13	45.15
*Weissella cibaria*	AB3b^b^	–	–	–	JWHV00000000	2,458,770	2321	–	7	62	1^c^	27.83	919	0.943	86.8	45.66	44.68
*Weissella cibaria*	ff3PR^b^	–	–	–	JWHT00000000	2,357,128	2178	–	7	64	1^c^	25.48	937	0.924	86.6	45.86	44.86
*Weissella cibaria*	MG1^b^	–	–	–	JWHU00000000	2,430,822	2238	–	4	57	1^c^	25.91	940	0.920	86.5	45.77	44.75
*Weissella confusa*	LBAE C39-2^b^	–	–	–	CAGH00000000	2,284,024	2097	–	8	66	1^c^	27.82	946	0.918	86.9	45.79	44.79
*Weissella halotolerans*	DSM 20190^b^	–	–	–	ATUU00000000	1,358,385	1314	–	13	59	1^c^	17.96	916	0.967	88.6	43.75	43.06
*Weissella paramesenteroides*	ATCC 33313^b^	–	–	–	ACKU00000000	1,962,173	1917	–	3	63	1^c^	23.19	888	0.976	86.8	38.75	37.88
*Weissella oryzae*	SG25^b^	–	–	–	BAWR00000000	2,129,279	2143	–	7	70	1^c^	29.49	852	1.006	85.8	39.95	38.90
*Weissella thailandensis*	fsh4-2^b^	–	–	–	HE575133-HE575182	1,968,992	1,8924	–	4	66	1^c^	18.84	899	0.961	86.5	39.66	38.74
*Weissella hellenica*	Wikim14^b^	–	–	–	BBIK00000000	1,915,620	1858	–	17	68	1^c^	19.96	884	0.969	85.8	37.46	36.61

^a^Previously described by Costa et. al. [14]

^b^Draft genomes. Except for NC36, all other draft genomes had no annotation and were submitted to RAST

^c^Not previously identified. Predicted in this work using the genome fasta file in ARAGORN v1.2.36 [70]

### Prediction of clusters of orthologous genes

The software orthoMCL was used to predict the clusters of orthologous genes using the Markov clustering (MCL) approach [22]. Basically, .faa files containing the amino acid sequences derived from all the coding sequences (CDSs) in each genome were exported from .gbk files, concatenated, and adjusted using orthoMCL scripts. A BLASTp analysis was applied to the resulting concatenated file against itself, with an e-value of 10^−20^, to generate an all-vs-all BLAST file. The all-vs-all BLAST file was loaded into the databases of orthoMCL and the sequences were clustered with the MCL software to generate the final groups of orthologous genes. In this analysis, CDSs shared by all strains were considered to be part of the core genome, whereas CDSs harbored by only one strain were considered to be singletons or strain-specific genes.

### Gene synteny analysis

The Mauve program was used to determine the gene synteny between the genomes of the *W. ceti* isolates. Mauve performs orthology comparisons between genomes to predict syntenic blocks, which reveals the rearrangement events between the genomes [23]. Here, progressiveMauve was used with the standard parameters. The contigs of *W. ceti* NC36 were ordered according to the genome synteny of the other strains (WS08, WS74, and WS105), before their analysis with Mauve, for easy visualization.

### Prediction of polymorphic sites

The polymorphic sites between the genomes of the *W. ceti* strains were analyzed with the whole-genome multilocus sequence typing (wgMLST) methodology using the gene-by-gene approach in the BIGSdb software, installed in a local server [24, 25]. Briefly, we first updated the BIGSdb database with the nucleotide sequences of all CDSs from the genome of *W. ceti* WS105, defined a scheme called “All_Loci”, and searched for the presence/absence and variant alleles of each CDS against the genomes of *W. ceti* strains WS08, WS74, WS105, and NC36.

### Prediction of *W. ceti* evolutionary pattern

The pattern of *W. ceti* evolution was determined by calculating Tajima’s D values and the dN/dS ratios for orthologous genes using the DnaSP software [26]. Briefly, the amino acid sequences derived from all CDSs were analyzed with the BLASTClust software (BLAST suite of software at the National Center for Biotechnology Information [NCBI]), using the standard parameters, to predict orthologous genes. The amino acid sequences were imposed to their nucleotide sequences counterparts, which were globally aligned using the Muscle software [27] with the standard parameters, and then concatenated and used as the input for DnaSP.

### Phylogenetic tree and networks construction

The phylogenetic networks for each of the datasets generated in the previous steps were constructed with the SplitsTree4 software [28]. Briefly, a more stringent core genome subset was retrieved from orthoMCL using nucleotide sequences with an e-value of 10^−20^, clustered with Muscle and analysed in SplitsTree4 using “parsimonysplits”. Also, one distance matrix was exported from Gegenees in the “nexus” format for use as the input into SplitsTree4. The distance matrix contained the percentage similarities of the all-vs-all genomes with a threshold of 40 %. The equal angle method was used to construct the phylogenetic network from the distance matrix generated by Gegenees. The final network was plotted with NeighborNet. Also, amino acid content and variability of hemolysins were analysed. For this task, the nr database at NCBI was searched with BLASTp using the sequences of all the hemolysin and hemolysin-like CDSs from the genus *Weissella*. The best hits were retrieved for global alignment with ClustalW2 [29]. The final distance matrix created with ClustalW2 was then used with the neighbor-joining method to construct the phylogenetic tree and the final tree was plotted as a phylogram.

Additionally, *W. ceti* strains were analysed using the wgMLST methodology of BIGSdb and a multiple alignment of the “All_loci” alleles was exported in .xmfa file format. The multiple alignment was then analysed in SplitsTree4 using “parsimonysplits” with 500 bootstraps to create a phylogenetic network.

### *In silico* prediction of genomic islands and phage sequences

Genomic islands (GEIs) were predicted with the Genomic Island Prediction software (GIPSy; http://www.bioinformatics.org/groups/?group_id=1180), choosing the option for the prediction of pathogenicity islands (PAIs). GIPSy updates the methodology of the previously published software, PIPS [30], which predicts putative PAIs by searching for regions larger than 6 kb that show genomic signature deviations (i.e., deviations in G+C content or codon usage), transposase genes, virulence factors, and flanking tRNAs. Additionally, it also checks for the absence of the target region from closely related species [30]. The putative GEIs, and more specifically PAIs, for *W. ceti* strains WS08, WS74, WS105, and NC36 were predicted using the *W. koreensis* KACC 15510 genome [GenBank: CP002899] as the nonpathogenic, closely related reference organism of the same genus. Putative phage sequences were predicted in the genomic sequences of *W. ceti* strains WS74 and WS105 using PHAST [31], and then GLAM2 (Gapped Local Alignment of Motifs) was used to identify the conserved attachment sites for the predicted phages [32].

### Construction of circular genomic maps

Circular genomic maps were created using the BRIG software [33]. Here, we used the GenBank files of the genomes of *W. ceti* strains WS08, WS74, WS105, and NC36 as the references and the genetic coordinates generated by GIPSy to plot the GEIs on the final circular genomic maps. For *W. ceti* NC36, we also ordered the contigs according to the genome synteny of the other strains (WS08, WS74, and WS105) before plotting the figure, for easy visualization.

### Identification of tandem repeat sequences in adhesins

The identification of tandem repeat sequences was performed with the software tandem repeats finder [34] using the whole-genome sequence of all *W. ceti* strains in fasta format. The tandem sequences were then mapped to the genbank annotated file and the regions overlapping adhesins were compared between all four *W. ceti* genomes. Also, all groups of orthologs of adhesins in the four genomes were analysed in the online software WDAC (Weighed Domain Architecture Comparison Tool) [35] to search for the presence of well-characterized repeated domains.

## Results and discussion

### General features

The general features of the *Weissella* genomes evaluated here are presented in Table 1. Briefly, the final genomes ranged in size from ~1.35 to ~1.42 Mb, whereas the draft genomes varied from ~1.35 to ~2.45 Mb. According to these sizes, the numbers of CDSs also varied between the final genomes (1269–1338) and the draft genomes (1258–2321).

In the species *W. ceti*, strains WS08, WS74, and WS105 were all isolated from outbreaks in Brazil on different farms in 2008, 2010, and 2012, respectively, and display different PFGE patterns, whereas NC36 was isolated in the USA. The WS08 and NC36 genomes are ~1.35 kb, whereas those of WS74 and WS105 are ~1.39 kb (Table 1). All four genomes have almost identical coding percentages and G+C contents in their genes and genomes. In general, the four strains show small variations in their numbers of CDSs, mean gene lengths, and gene densities, which arise from differences in their genome sizes. The only major differences are related to the number of rRNAs and tRNAs encoded, with fewer in NC36. Because rRNAs, tRNAs, and other repetitive sequences are recognized as problematic regions in genome assembly, the draft status of the NC36 genome may explain the discrepancy in the numbers of these noncoding regions between the Brazilian and American strains of *W. ceti*.

### Prediction of commonly shared and species-specific genes of *Weissella* species

**Fig. 1:**
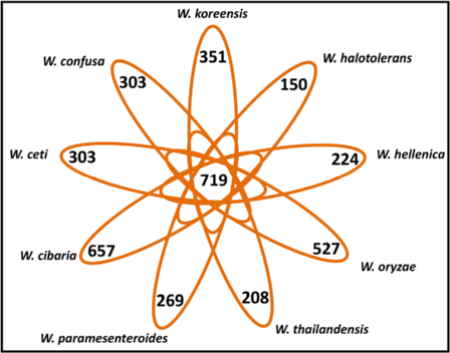
Schematic view of the core genes and singletons of all *Weissella* species. The number in the center represents the number of core CDSs shared by all species, whereas the number on each branch indicates the number of singletons carried by each species

To predict the set of commonly shared and species-specific genes of all the species in the genus *Weissella*, we used orthoMCL to define the clusters of orthologous genes [22]. The CDSs distributed throughout all species were defined as parts of the core genome, whereas those that were present in only one strain were defined as singletons or strain-specific genes. In total, 719 CDSs were shared by all species of *Weissella* (Fig. 1). Most were involved in basic cell functions and were classified under the “information storage and processing” and “metabolism” classes in the clusters of orthologous groups (Additional file 4). Interestingly, the longest genomes in the dataset, represented by *W. cibaria* (~2.31–2.45 Mb), *W. confusa* (2.28 Mb), and *W. oryzae* (~2.12 Mb), had 657, 303, and 527 singletons, respectively, whereas the species *W. koreensis* and *W. ceti*, with genomes of ~1.35–1.72 Mb, have 351 and 303 singletons, respectively. In contrast, in an intraspecies analysis, WS74 and WS105 presented with 41 and 42 singletons each, whereas NC36 and WS08 had only 7 and 4 singletons, respectively, which indicates a high-similarity in the genetic content among the *W. ceti* strains analyzed.

### Phylogenomic and comparative genomic analyses

**Fig. 2:**
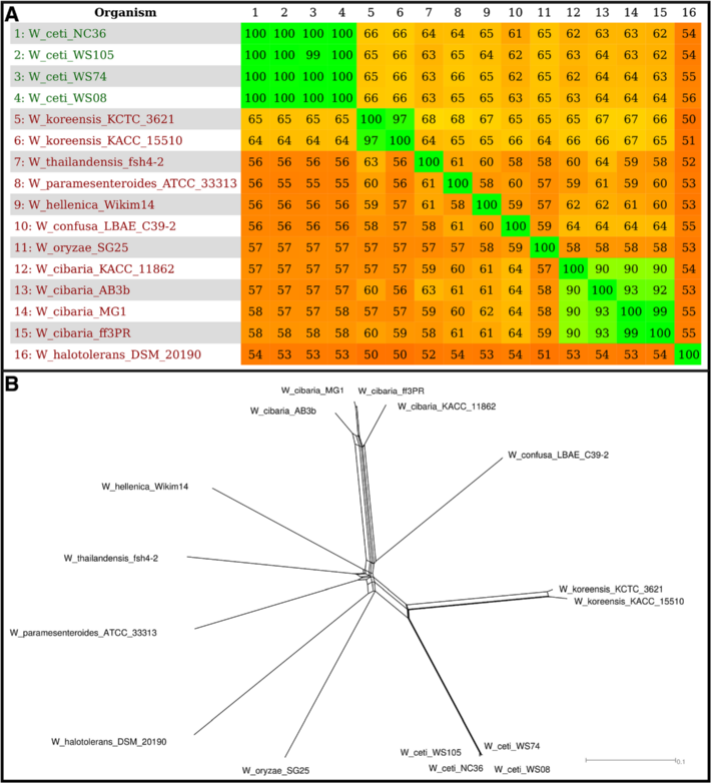
Heatmap and distance-matrix-based phylogenetic network of the genus *Weissella*. **a** Species on the left side of the figure are represented in the same numeric order above the figure. The numbers show the percentage similarity between the conserved regions of the genomes, where the colors vary from orange (low similarity) to green (high similarity); **b** NeighborNet-plotted network using the equal angle method, with the distance matrix from Gegenees as the input

To determine the degree of genomic variability between *W. ceti* and the other species in the genus *Weissella*, we performed a comparative genomic analysis using the Gegenees software and plotted the resulting distance matrix as a heatmap. According to the heatplot generated with Gegenees (Fig. 2a and Additional file 5), interspecies similarity varied from ~50 % between *W. koreensis* KCTC 3621 and *W. halotolerans* DSM 20190 to ~68 % between *W. koreensis* KCTC 3621, *W. paramesenteroides* ATCC 33313 and W. thailandensis fsh4-2. *W. koreensis* was the species that presented the highest degree of similarity against *W. ceti*, ranging from ~64 to ~66 %. In contrast, the *W. ceti* strains displayed a high degree of intraspecies similarity, ranging from ~99 % between WS105 and WS74 to ~100 % between all other strains, whereas *W. koreensis* strains KACC 15510 and KCTC 3621 displayed intraspecies similarity of ~97 %. The *W. cibaria* strains displayed the lowest degree of intraspecies similarity, ranging from ~90 % between KACC 11862 and all other *W. cibaria* strains to ~99 % between the strains MG1 and ff3PR.

**Fig. 3:**
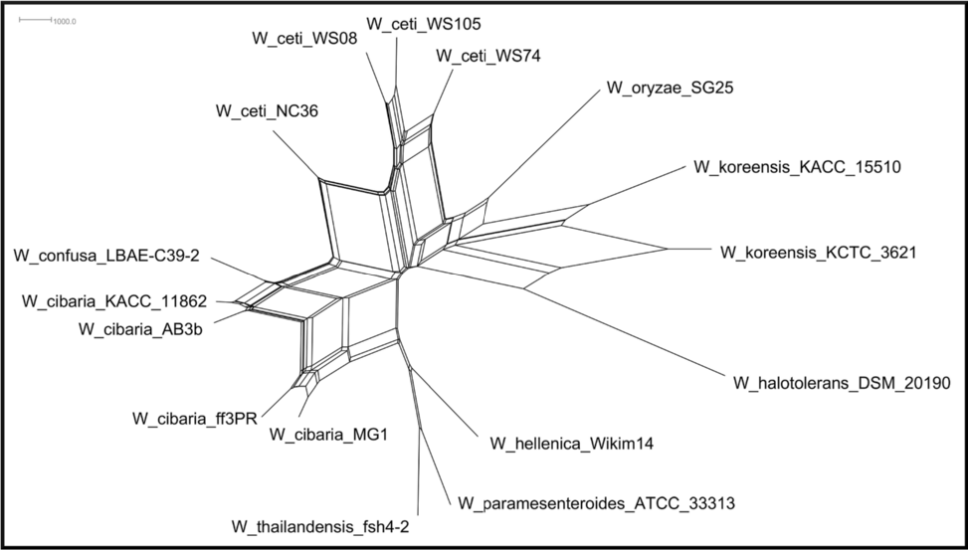
Phylogenetic relationship of *Weissella* using the multiple alignment of nucleotide core genome from orthoMCL. The network was generated with splitstree software using “parsimonysplits”

We also used the distance matrix generated with Gegenees, from the evolutionary distance based on the similarity between the strains, to determine how well the similarity heatplot correlated with the phylogenetic relationships reported in the literature [1, 12]. On this phylogenetic network, the *Weissella* species clustered in two separate groups: one including *W. ceti* and *W. koreensis*, and the other including *W. hellenica*, *W. cibaria*, *W. confusa*, *W. paramesenteroides*, *W. thailandensis*, W*. halotolerans*, and *W. oryzae*. The network generated with Gegenees is consistent in the clustering of *W. cibaria* and *W. confusa* with previously reported phylogenetic trees based on 16S rDNA, whole-cell protein patterns, and *Cla*I, *Eco*RI, and *Hin*dIII ribopatterns (Fig. 2b and Additional file 5) [1, 12]. It also shows that *W. paramesenteroides* is closely related to the *W. confusa* and *W. cibaria* cluster, whereas *W. halotolerans* is more distantly related to this cluster, which is consistent with all previously reported phylogenetic trees, except the one created with *EcoRI* ribopattern [1, 12]. Also, we have generated another phylogenetic network from the alignment of a more stringent core genome predicted using orthoMCL with nucleotide sequences of all *Weissella* strains (Fig. 3 and Additional file 5). Except for the clustering of *W. halotolerans* and *W. oryzae* with *W. koreensis*, all other relationships were maintained, with *W. paramesenteroides* closely related to *W. confusa* and *W. cibaria*, whereas *W. halotolerans* is more distantly related. Also, *W. koreensis* and *W. ceti* are closely related. Although the *W. ceti* strains have a high-similarity at the genetic content, they may still have some small differences at the nucleotide level, which would not be tracked by Gegenees, as highlighted by the core genome phylogenetic analysis. To identify those differences at the nucleotide level, we performed a polymorphism-based phylogenetic analysis of the *Weissella* genomes.

### Deeper view of the high-similarity of *W. ceti* using wgMLST

**Fig. 4:**
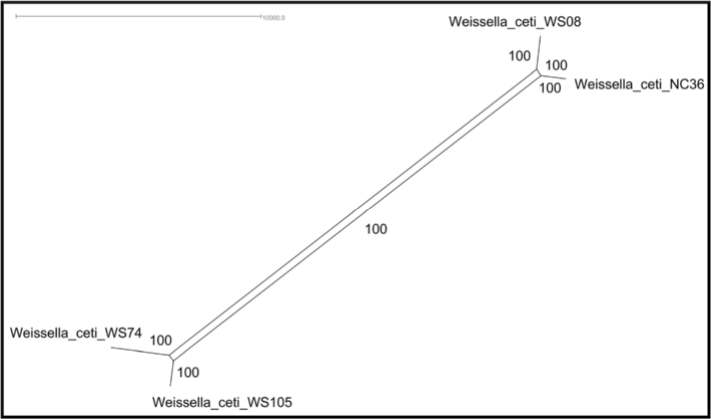
Phylogenetic network depicting the relationships of all *Weissella ceti* strains based on polymorphic loci. Phylogenetic network plotted using the parsimonysplits with 500 bootstraps, with the multiple sequence alignments from All_loci

Polymorphisms have been widely identified with conventional MLST analyses based on a few housekeeping genes. However, considering that allele changes are single genetic events, MLST can miss major horizontal gene transfer (HGT) events, which are extremely important for the differentiation of strains. In view of this limitation of MLST and the ever-growing genomic data deposited in databases, a new gene-by-gene approach has been successfully used to discriminate taxa from inter- to intraspecies levels with high resolution, even allowing the discrete genetic variability in different strains isolated from a single patient to be tracked [25]. In these analyses, a larger number of tracked loci allows higher resolution in intraspecies analyses based on whole genome sequences with wgMLST. Given the high-similarity at the genetic content of *W. ceti*, we sought to create a phylogenetic network with better resolution at the intraspecies level than the one achieved with Gegenees, using the wgMLST methodology with a gene-by-gene approach in BIGSdb (Fig. 4 and Additional file 5).

On the All_loci-based phylogenetic network (Fig. 4 and Additional file 5), strains WS74 and WS105 were more closely related to each other than to WS08 or NC36 (Fig. 4 and Additional file 5). This result shows an interfarm variation in the Brazilian isolates, and more interestingly, a close relationship between the strain from one of the first outbreaks in Brazil and the American isolate. From this perspective, we cannot correlate the strains with their places of origin, because the Brazilian strains did not cluster separately from the American strain.

We applied Tajima’s D method to determine whether these strains were under different mutational pressures. According to Tajima’s D, a highly positive D value indicates balancing selection, with few rare variants, whereas a strongly negative D value results from an abundant number of rare variants, indicating purifying selection [36]. This analysis can be applied to coding regions and also extrapolated to synonymous and nonsynonymous regions. The ratio of the D value calculated for the nonsynonymous regions (dN) and the D value calculated for the synonymous region (dS) is indicative of positive (dN/dS >1), purifying (dN/dS <1), or neutral selection (dN/dS = 1) [37, 38].

The D values for the *W. ceti* strains (*n* = 4) were calculated using global codon alignments generated for the orthologous genes shared by the genomes in the dataset. The D values for coding regions of the *W. ceti* strains were very low, in the order of −0.795 (not significant according to [36]), whereas the dN/dS ratio was 1.2048. Although this dN/dS ratio (>1) indicates slightly positive selection, the D value indicates rather neutral evolution, in which the polymorphisms are driven only by stochastic mutations and genetic drift.

### Genomic synteny and gene conservation among *W. ceti* strains

**Fig. 5:**
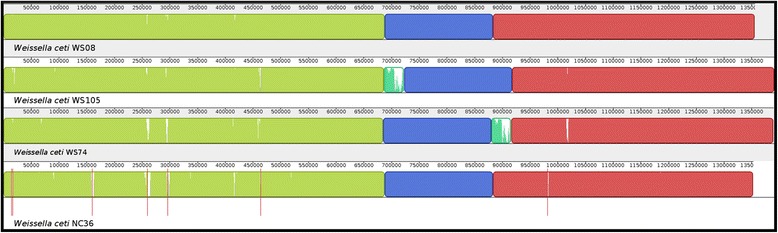
Genomic synteny and gene conservation between the four strains of *Weissella ceti*. From top to bottom: strains WS08, WS105, WS74, and NC36. *Weissella ceti* strain WS08 was used as the reference genome. The contigs of *W. ceti* strain NC36 were reordered before plotting. Blocks with same color represent large syntenic regions between the genomes, whereas the white portions inside the blocks are regions of low similarity. Red vertical bars delimit the contigs

Although highly similar in their gene content, different strains of the same species may display gene rearrangements that allow them to develop different traits. To determine whether this is the case for the *W. ceti* strains, we used the Mauve software to plot the gene synteny between *W. ceti* strains WS08, WS105, WS74, and NC36 (draft genome). All strains displayed a high degree of synteny, as shown by the order of the syntenic blocks (Fig. 5). It is noteworthy that the genomes of strains WS105 and WS74 both contain an inserted region (light green in Fig. 5) of ~36 kb at positions 686–722 kb and 880–916 kb, respectively. These regions are approximately the same size and partially share ~50 % of their sequences, as shown by the light green peaks inside the box (Fig. 5). We investigated whether these regions were PAIs, which could account for the putative differences in the pathogenesis and/or adaptability of strains WS105 and WS74 compared with the other strains, as discussed in the next section.

### Putative pathogenicity islands

HGT plays a pivotal role in bacterial evolution in the adoption of new traits and adaptation to new hosts. In this context, GEIs are very important because they can incorporate a large number of genes in a single event, allowing bacteria to gain multiple new traits and traits requiring many genes such as secretion systems [39, 40].

**Fig. 6:**
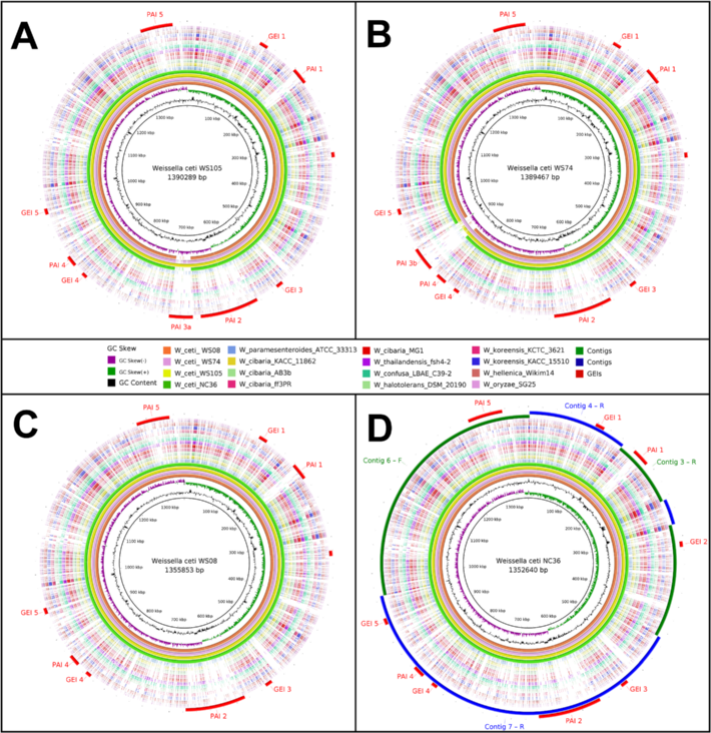
Circular genome comparison of *Weissella ceti* genomes showing the locations of putative GEIs. **a, b, c,** and **d** were plotted using *W. ceti* strains WS105, WS74, WS08, and NC36 as the references, respectively; GEIs, putative genomic islands; Contigs, indicates the contigs, in two different rings, for easy visualization. The contigs of *W. ceti* NC36 are ordered according to the genome of WS08, where F and R, after the contig numbers, indicate their orientation: forward and reverse complement, respectively

We used the GIPSy software to identify putative PAIs in *W. ceti*, using the genome sequence of *W. koreensis* KACC 15510 as the nonpathogenic, closely related reference species [30]. Briefly, *W. koreensis* KACC 15510 was isolated from Chinese cabbage kimchi, a Korean fermented food, which contains diverse groups of LAB and is recognized for its health-promoting characteristics [41]. GIPSy prediction identified 10 putative GEIs (GEIs 1–5 and PAIs 1–5) in *W. ceti*, which were distributed throughout the genome sequence, with lengths that varied from ~7.6 kb (GEI 2) to ~89.4 kb (PAI 2). It is noteworthy that PAIs 2 and 5 are partially absent from the other species of the genus *Weissella*, whereas PAIs 1 and 3 are completely absent from all other species, i.e., they are species-specific *W. ceti* PAIs (Fig. 6). PAI 3 is also absent from WS08 and NC36, and occurs in the same rearranged light-green region of WS74 and WS105 shown in the Mauve gene synteny analyses. PAI 3 has a different composition in WS105 and WS74, and has therefore been designated PAI 3a and PAI 3b, respectively. Among the 302 genes carried by all 10 GEIs, 140 (~46 %) were annotated as hypothetical proteins, which is far higher than the genomic mean (~21–24 %) shown in Table 1. In view of this high number of uncharacterized genes, we focused on the PAIs that were absent from the other species of *Weissella*, and that harbouring genes encoding proteins with putative prominent functions related to known virulence mechanisms, such as PAIs 1, 2, 3a, and 3b.

**Fig. 7:**
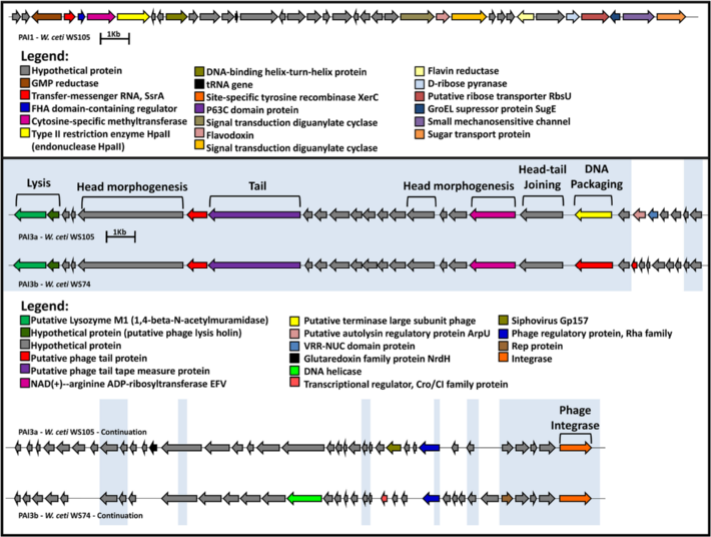
Gene content of the two species-specific PAIs of *Weissella ceti*. On top, PAI 1; on bottom, PAIs 3a and 3b; regions highlighted in blue represent genomic sequences shared by both PAIs 3a and 3b. Brackets over the figure show the phage regions: lysis, head morphogenesis, tail, head-tail joining, DNA packaging, and phage integrase. For ease of representation, the intergenic regions are not to scale

**PAI 1** – The *ssrA* gene (WS105_tm01) from PAI 1 (Fig. 7) is putatively transcribed into a hybrid transfer–messenger RNA (tmRNA) [42], whereas the gene encoding the cofactor SmpB (WS105_0199), an SsrA-binding protein, is located elsewhere in the genomic sequence. Those two genes are widely conserved in all species of *Weissella* described here. tmRNA, in association with cofactor SmpB, plays a pivotal role in rescuing stalled ribosomes in bacteria by providing a stop codon *in trans*, in a process called “trans-translation” [43–45]. Interestingly, studies of avian pathogenic *Escherichia coli* and *Francisella tularensis* have shown that this trans-translational process plays an important role in their resistance to diverse stress conditions and in the virulence of these pathogens [43, 45, 46]. However, given the ubiquity of tmRNA-smpB system in bacteria [47, 48] the presence of the only copy of a tmRNA gene in *W. ceti* inside PAI 1 and the absence of an alternative ribosome-rescue system (*arfA* and *arfB*) in the genome require further study before this pattern can be correlated with the virulence of *W. ceti*.

**Fig. 8:**
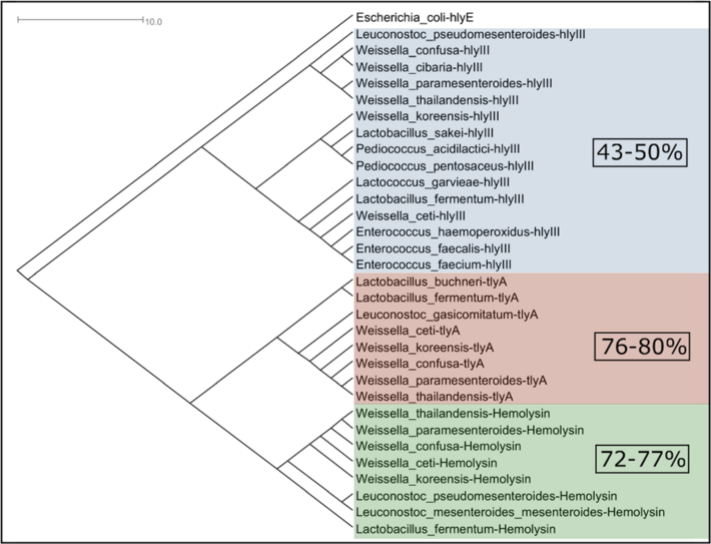
Phylogenetic tree depicting the degrees of homology between the *Weissella* species and other genera in their hemolysin III, hemolysin A, and hemolysin-like proteins. Colors depict different orthologous genes. In red, *tlyA*, a hemolysin-A-encoding gene; in blue, *hlyIII*, a gene encoding hemolysin III; and in green, “hemolysin” indicates a hemolysin-like protein. The percentages represented inside the figure are the ranges of similarities between the gene carried by *W. ceti* and its counterparts in other *Weissella* species

**PAI 2** – *Weissella confusa* and *W. ceti* both display α-hemolytic profiles at 35 °C and 37 °C, respectively [7, 14]. These profiles may possibly be attributable to the presence of a shared *tlyA* gene encoding an α-hemolysin in the genomes of *W. confusa* (WEISSC39_09830) and *W. ceti* (WS105_0965). Both species also harbor two additional genes encoding hemolysins: hemolysin III (*hlyIII*, WS105_0554, annotated as hypothetical protein) and a hemolysin-related protein (WS105_0227, annotated as hypothetical protein). Interestingly, the *hlyIII* gene of *W. ceti* is harboured by PAI 2, which is absent from all other *Weissella* species, suggesting that the *hlyIII* genes of *W. ceti* and *W. confusa* were acquired by both species during different evolutionary events. To determine whether these genes are similar at the amino acid level, we have searched for sequences with similarities to all three hemolysins in *Weissella* species using BLASTp, retrieved the sequences, aligned them with UniProtKB, and generated phylogenetic tree for easy visualization of the comparison (Fig. 8). From the phylogenetic tree, TlyA and the hemolysin-related proteins of *W. ceti* display amino acid identities of 76 %–80 % and 72 %–77 %, respectively, to those of the other *Weissella* species, whereas the HlyIII protein of *W. ceti* displays lower identities, of 43 %–50 %, with those of other *Weissella* species, but a high identity of 72 % with *Enterococcus faecalis* hemolysin. These differences in amino acid sequences between the HlyIII proteins encoded by *W. ceti* and other *Weissella* species and the close relationship between this protein with its counterparts in bacteria from other genera support the suggestion that this gene was acquired by *W. ceti* through HGT. Also noticeable, the HlyIII protein of *W. ceti* is highly similar to the one harboured by *Lactococcus garviae*, a classic fish pathogen, which inhabits the same aquatic environment and host as *W. ceti*.

Hemolysins belong to a family of bacterial virulence factors, the pore-forming cytotoxins (PFTs) [49, 50]. One of the most prominent and well-characterized PFTs is α-hemolysin from *Staphylococcus aureus* (Hla, also known as α-toxin) [51, 52]. In *S. aureus*, the expression of Hla is tightly controlled by the accessory gene regulator (*agr*) locus, a quorum-sensing (QS) system that regulates the expression of specific virulence genes in a coordinated and temporal fashion [53–55]. Interestingly, *W. ceti* contains genes encoding the two-component system regulators, AgrA (WS105_0510, annotated as LytTr DNA-binding domain protein) and AgrC (WS105_0511, annotated as Sensor protein CitS), which, according to the orthoMCL clusters of orthologous genes, are species-specific genes, i.e., they are not present in any other *Weissella* species. A two-component system with the same *agrAC* structure has also been reported to function in other species [56]. However, additional experiments are required to clarify whether hemolysin III and the *agr* operon are expressed and functional in *W. ceti*.

**PAIs 3a and 3b –** PAIs 3a and 3b contain a lysozyme M1 gene (WS105_0603), encoding a 1,4-β-N-acetylmuramidase (Fig. 7), which has orthologous counterparts in *W. hellenica* and *W. paramesenteroides*. 1,4-β-N-Acetylmuramidase plays a pivotal role in bacterial lysis, allowing the extrusion of progeny phage [57]. Moreover, lysozymes do not have signal peptides or any other membrane-targeting system, but access the membrane structure through the action of holin molecules inserted into the cytoplasmic membrane [57]. To cope with lysozymes and other antibacterial peptides produced by immune cells, Gram-positive and Gram-negative bacteria have adapted their peptidoglycan structures to avoid degradation [58]. One such mechanism of peptidoglycan modification in *S. aureus* is the O-acetylation of the C-6 position in NAM by O-acetyltransferase A (encoded by the *oatA* gene) [58]. Interestingly, as well as encoding copies of lysozyme M1 inside PAIs 3a and 3b, *W. ceti* also encodes another lysozyme M1 in PAI 4 (WS105_0837, annotated as ToxA_2 protein), a holin lysis gene in PAIs 3a and 3b (WS105_0604) (Fig. 7), and an *oatA* gene in PAI 2 (WS105_0570).

Because PAIs 3a and 3b contain many phage-related genes, we used the PHAST software to predict putative phage sequences inserted into the *W. ceti* genome, using the genome sequences of strains WS105 and WS74. PHAST predicted one possible phage sequence inserted into the genome of *W. ceti* WS105, represented by PAI 3a, and one intact phage in WS74, represented by PAI 3b. We propose that PAIs 3a and 3b were acquired as complete intact phages, containing i) the lysis portion of the phage structure, which is composed of lysozyme M1 and holin genes, as described in the previous section; ii) the head morphogenesis sequence; iii) the head–tail joining sequence; iv) the tail sequence; v) the DNA packaging sequence; and vi) the phage integrase sequence, which is probably responsible for the incorporation of the whole phage region (Fig. 7). The two intact phages also seem to have been incorporated into the genomes of strains WS105 and WS74 based on the recognition of two different attachment sites, with the motifs 1 and 2, respectively, showed in Additional file 6. These also support the presumption of two independent genomic transfer events.

From these analyses, it can be argued that *W. ceti* evolved from an ancestral species by the incorporation of long PAIs, which allowed the bacteria to adopt new traits and to adapt to new hosts. The absence of PAIs 3a and 3b from strains WS08 and NC36, together with the differences between these two PAIs, their conservation of only those genes encoding phage structural proteins, and the presence of different flanking insertion sequences all suggest that both PAIs 3a and 3b were incorporated during very recent and independent HGT events, rather than by transfer from an unique ancestral genome.

### Adhesion

Except for the incorporation of PAIs 3a and 3b into *W. ceti* WS105 and WS74, respectively, and the absence of some rRNA genes in the draft genome of *W. ceti* NC36, the only major differences between all the *W. ceti* strains sequenced so far are located in the collagen adhesins, platelet-associated adhesin, and mucus-binding protein. These adhesins are included among the singletons of *W. ceti* (species-specific genes) and were identified in all four strains of *W. ceti* analyzed here (Table 2), including *W. ceti* NC36 [17]. Except for the CDS WCNC_00912, encoding the collagen adhesin precursor, all other CDSs (WCNC_00917, WCNC_00922, WCNC_05547, WCNC_06207, WCNC_01820, and WCNC_01840) are structurally different in the other *W. ceti* strains. Briefly, two CDSs encoding collagen adhesins in NC36 (WCNC_00917 and WCNC_00922) are merged into one single CDS in strains WS08, WS74, and WS105; another two CDSs of NC36, also encoding collagen adhesins (WCNC_05547 and WCNC_06207) are longer in the other strains of *W. ceti*, spanning the regions in which the orthologues of WCNC_05542 and WCNC_06202 should be located, respectively. The sequence encoding platelet-associated adhesin, WCNC_01820, is also longer in the other strains of *W. ceti*, containing the regions in which the orthologues of WCNC_01825 and WCNC_01830 should be located. The gene encoding the mucus-binding protein, WCNC_01840, is also longer in the other *W. ceti* strains, spanning the region in which the orthologue of WCNC_01835 should be located.

**Table 2:** Putative collagen, mucus-binding, and platelet-associated adhesins encoded by *Weissella ceti*

WS08	WS74	WS105	NC36	Product	Score	Most-similar orthologue
WS08_0070	WS74_0069	WS105_0070	WCNC_00922	Collagen adhesin protein (annotated as: Hypothetical protein)	99.4–100 %	*Enterococcus faecalis* EnGen0301– 33 % id - 99 % query cover
			WCNC_00917			
WS08_0071	WS74_0070	WS105_0071	WCNC_00912	Collagen adhesin precursor	100 %	*Enterococcus faecalis* – 29 % id – 97 % query cover
WS08_0360	WS74_0360	WS105_0358	WCNC_01820	Hypothetical protein	96.3–100 %	*Staphylococcus pasteuri* – 37 % id – 23 % query cover
			WCNC_01825			
			WCNC_01830			
WS08_0361	WS74_0361	WS105_0359	WCNC_01835	Mucus-binding protein (annotated as: internalin-J precursor)	98.4–99.9 %	*Lactobacillus salivarius* - 37 % id - 73 % query cover
	WS74_0362		WCNC_01840			
WS08_0450	WS74_0451	WS105_0448	WCNC_06207	Collagen adhesin precursor (annotated as: Hypothetical protein)	99.9–100 %	*Listeria aquatica* FSL S10-1188 - 38 % id - 16 % query cover
			WCNC_06202			
WS08_0583	WS74_0584	WS105_0581	WCNC_05547	Collagen adhesin precursor	99.9–100 %	*Enterococcus faecalis* - 27 % id - 98 % query cover
			WCNC_05542			

Additionally, we sought to find whether the variations in size of the adhesins were related to a variable number of tandem repeat sequences, a common feature of surface proteins from fungi, bacteria and other pathogens [59, 60]. For this task, we have used the software tandem repeats finder [34] and compared the orthologs of each adhesin described in Table 2 using the online software WDAC [35]. From the six groups of orthologs, we have found tandem repeat sequences in all adhesins, where only WS08_0071 and WS08_0583 present fixed numbers of tandem repeats. However, although the other sequences present variations in the number of tandem repeats, only WS08_0361 and its orthologs presented variable numbers of a well-characterized domain, MucBP domain: six in WS74; nine in WS105 and NC36; and, ten in WS08 (Additional file 7).

Bacterial infection involves a cascade of events, and adhesion to the host tissue is the first critical step in the pathogenic process. It is usually mediated by a multitude of cell-wall-anchored proteins and assembled protein structures. These assembled protein structures are mainly represented by pili or fimbriae that protrude from the cell, whereas other single-molecule bacterial adhesins specifically bind to the host extracellular matrix components (such as fibronectin, collagen, fibrinogen, and others) and are collectively designated “microbial surface components recognizing adhesive matrix molecules” (MSCRAMMs) [61, 62]. Sortases play a pivotal role in anchoring MSCRAMMs the cell-wall by specifically recognizing the conserved LPxTG motif [62]. In *W. ceti*, there is a housekeeping sortase (WS105_0911), which is highly similar to the sortases of *W. cibaria*, *W. halotolerans*, *W. oryzae*, and *W. confusa*. Moreover, the gene *sraP*, which encodes a platelet-binding protein that forms a fimbria-like structure involved in adhesion, is normally organized in an operon with genes encoding a specific secretion (sec) system (SecA2, SecY2) and a glycosyltransferase, which are responsible for the translocation and glycosylation, respectively, of the SraP protein [63]. However, in *W. ceti*, the *sraP* gene is not organized in the same operon structure, and the only sec machinery genes are the canonical ones, i.e., the genes encoding the cytoplasmic preprotein translocase subunit SecA (WS105_1073) and the SecY/SecE/SecG protein-conducting pore proteins (WS105_1140, WS105_1121, and WS105_0197, respectively). In the absence of the SecA2–SecY2 secretion system, the putative translocation of the proteins encoded by the *sraP* genes and their role in the pathogenesis of *W. ceti* remain to be clarified with in vitro techniques.

### Antibiotic-resistance-related mechanisms of *W. ceti*

In the first case of *W. ceti* infection described in the rainbow trout *Oncorhynchus mykiss* in China, the species was shown to be resistant to several antimicrobials [10]. Seventy-seven strains isolated from diseased rainbow trout in Brazil were all resistant to sulfonamide and susceptible to florfenicol, and one of these strains was also resistant to erythromycin, two to oxytetracycline, and three to norfloxacin. WS08 and WS74 were also isolated among these 77 strains and are resistant to sulfonamide, but are currently susceptible to the other four antibiotics assayed (florfenicol, erythromycin, norfloxacin, and oxytetracycline) [11]. Interestingly, all the sequenced strains of *W. ceti* carry a gene putatively encoding a bicyclomycin/sulfonamide-resistance protein (*bcr*), which behaves like the permeases of the major facilitator superfamily (MFS), corroborating the previously reported antibiotic profiles of these strains (Additional file 8). They also carry a fosfomycin-resistance gene (*fosB*), a multiple-antibiotic-resistance regulator (*marR*), and several other CDSs that are similar to MFS-encoding genes. MFS is a family of transport systems, also called the uniporter–symporter–antiporter family, that includes transporters of a variety of small solutes, including drug efflux pumps [64].

### Adaptation of *W. ceti* to cold temperatures

*Weissella ceti* is a mesophilic bacterium and the strain isolated from the beaked whale is reported to grow in culture medium at low temperatures, such as 22 °C, but not at 15 °C [13]. In contrast, the rainbow trout, the main host of *W. ceti*, has a tolerance for temperatures ranging from 9 to 15 °C. Above this temperature, the fish usually displays progressive stress. In all outbreaks of weissellosis reported in China, Brazil, and the USA, an increase in water temperature up to 17 °C was described as a potential risk factor for the disease [11, 12, 14]. Therefore, the pathogenicity of weissellosis seems to be closely related to the ability of *W. ceti* to adapt to cold temperatures. To check this, we looked for heat-shock and cold-shock genes in the *W. ceti* genome and analyzed the ability of the Brazilian isolates to grow and survive at 15 °C.

Heat- and cold-shock responses are physiological mechanisms used by living cells to cope with high and low temperatures by expressing the so-called heat- and cold-shock proteins (HSPs and CSPs), respectively [65]. Although specific bacteria may be better fitted to particularly low, medium, or high temperatures, they have all evolved similar strategies to adapt to temperature variations. For instance, two orthologous CSP genes may be considered to be a cold-acclimation gene in a psycrophilic organism and a cold-shock gene in a mesophilic bacterium [66]. Briefly, CSPs act as RNA chaperones, binding mRNAs to prevent secondary folding, and thus facilitating their translation under cold-shock conditions [67]. In contrast, HSPs include chaperones and proteases with roles in protein folding, preventing protein denaturation under heat-shock stress. More interestingly, many HSP-encoding genes may also act in bacterial pathogenesis and survival inside macrophages [68], and may play important roles during cold-shock stress [69].

The *W. ceti* strains contain a *dnaJ*–*dnaK*–*grpE*–*hrcA* operon, which is probably involved in heat-shock resistance, a GroESL-encoding system, and three additional genes encoding cold-shock-related proteins, one of which (*cspC*) is located in PAI 4 (Table 3) and is shared with *W. halotolerans*, *W. thailandensis*, and *W. paramesenteroides*. Except for *cspC*, all other HSP- and CSP-encoding genes are highly conserved in *W. paramesenteroides*, *W. thailandensis*, *W. confusa*, *W. cibaria*, *W. halotolerans*, and *W. oryzae*. The presence of HSP- and CSP-encoding genes in *W. ceti*, even those shared with other *Weissella* species, may have played an important role in the adaptation of this bacterium to fish hosts, in which variations in water temperature could pose a highly adverse environment. The presence of larger numbers of rRNA operons in *W. ceti* than in the other *Weissella* species could also facilitate the maintenance of protein synthesis in the pathogen at adverse temperatures. However, the draft status of the genomes of most *Weissella* species allows the possibility that they carry additional rRNA copies that were missed during genome assembly.

**Table 3:** Putative heat- and cold-shock proteins encoded by *Weissella ceti*

WS08	WS74	WS105	NC36	Prokka annotation	Best blast hit on Uniprot database, ordered by Identity
WS08_0241	WS74_0241	WS105_0239	WCNC_00097	S4-domain protein	Ribosomal-RNA-binding protein; heat-shock protein
WS08_0769	WS74_0772	WS105_0833	WCNC_04647	Putative chaperone protein DnaJ	Molecular chaperone DnaJ
WS08_0770	WS74_0773	WS105_0834	WCNC_04642	Chaperone protein DnaK	Molecular chaperone DnaK; HSP70
WS08_0771	WS74_0774	WS105_0835	WCNC_04637	Protein grpE	Heat-shock protein GrpE
WS08_0772	WS74_0775	WS105_0836	WCNC_04632	Heat-inducible transcription repressor HrcA	Heat-inducible transcription regulator HrcA
WS08_0927	WS74_0993	WS105_0989	WCNC_02232	60 kDa chaperonin	Chaperonin GroEL
WS08_0928	WS74_0994	WS105_0990	WCNC_02237	10 kDa chaperonin	Co-chaperonin GroES (HSP10)
WS08_0262	WS74_0262	WS105_0260	WCNC_01320	Cold-shock protein CspB	Cold-shock protein CspB
WS08_0785	WS74_0788	WS105_0849	WCNC_04567	CspC protein	CspA family cold-shock transcriptional regulator
WS08_0850	WS74_0916	WS105_0913	WCNC_04242	DEAD/DEAH box helicase	ATP-dependent RNA helicase

Although Vela et al. [13] reported that *W. ceti* cannot grow at 15 °C, all the Brazilian strains of *W. ceti* (WS08, WS74, and WS105) grew at this temperature and were viable after incubation for 15 days in BHI. Taken together, these results suggest that the fish strains of *W. ceti* are adapted to grow at low temperatures.

## Conclusions

In this study, we undertook a comparative analysis of the four currently published genomes of *W. ceti* strains WS08, WS74, WS105, and NC36. According to phylogenomic analysis, the *W. ceti* strains isolated from different rainbow trout farms in Brazil and the USA present high degrees of similarity despite the lack of epidemiological linkage between farms and between countries. This same pattern can also be inferred from Tajima’s D, which revealed a pattern of neutral evolution, and from the synteny map, in which all the *W. ceti* strains showed highly homogeneous genome compositions. Also, we have predicted 10 GEIs across the genomes of the *W. ceti* strains, one of which (PAI 3) is only present in the genomes of WS105 and WS74. This was acquired through phage incorporation and has signs indicating two separate HGT events. *Weissella ceti* also carries an *oatA* gene in PAI 2, which probably accounts for its resistance to lysozyme, which could allow the bacterium to survive the lytic phage cycle, with the incorporation of the new phage sequences containing lysozyme-encoding genes. If we follow the necessary steps for a successful pathogenic process, *W. ceti* has genes putatively encoding proteins involved in: survival in the water environment under stressful temperatures (CSPs and HSPs); contact with the host cell (adhesins); cell lysis and bacterial spread inside the host (hemolysins and their regulators); resistance to immune-cell-mediated stresses (*tmRNA*, *oatA*, CSPs, and HSPs); and antibiotic resistance (sulfonamide-resistance protein and several multidrug efflux pumps). The analyses presented here provide some insight into the pathogenesis of this newly emerging pathogen and should drive new research into the host–pathogen interactions of *W. ceti*.

## Availability of supporting data

The data sets supporting the results of this article are included within the article and its additional files. Furthermore, the GenBank Accession Numbers of analyzed strains are shown in Table 1 and Phylogenetic data were deposited in TreeBase and are publicly available at http://purl.org/phylo/treebase/phylows/study/TB2:S18594.

## Additional files

Additional file 1:
**Sequencing information of** ***W. ceti*** **WS08.** (DOCX 10 kb) Additional file 2:
**Assembly information of** ***W. ceti*** **WS08.** (DOCX 10 kb) Additional file 3:
**PFGE and predicted genome sizes of** ***W. ceti*** **WS08, WS74 and WS105 calculated** ***in silico*** **with Bionumerics.** (TIFF 220 kb) Additional file 4:
**Core genes of** ***Weissella*** **genus classified by COG functional category.** (TIFF 80 kb) Additional file 5:
**Nexus files and align file of phylogenetic networks.** (ZIP 894 kb) Additional file 6:
**Phages attachment sites motifs.** (TXT 192 bytes) Additional file 7:
**Visual representation of the MucBP repeated domain in orthologs of WS08_0361 from** ***W. ceti*** **strains. The proteins with “*” were concatenated into one sole sequence before submission to WDAC.** (TIFF 512 kb) Additional file 8:
**Putative multidrug-efflux-related proteins of** ***Weissella ceti.*** (DOCX 17 kb)

## Abbreviations

BHI: brain-heart infusion broth
CDS: coding sequence
CSP: cold-shock protein
GEI: genomic islands
HGT: horizontal gene transfer
HSP: heat-shock protein
LAB: lactic acid bacteria
MCL: Markov clustering
MFS: major facilitator superfamily
MLST: multilocus sequence typing
MSCRAMMs: microbial surface components recognizing adhesive matrix molecules
PAI: pathogenic genomic island
PFGE: pulsed-field gel electrophoresis
PFT: pore-forming cytotoxins
PGM: Personal Genome Machine
QS: quorum-sensing
rRNA: ribosomal RNA
tmRNA: transfer–messenger RNA
tRNA: transport RNA
wgMLST: whole-genome multilocus sequence typing
