# Supplementary material for: Comparative genome analysis of Weissella ceti, an emerging pathogen of farm-raised rainbow trout
Source: BMC Genomics. 2015 Dec 22;16:1095. doi: 10.1186/s12864-015-2324-4 (PMC4687380; doi:10.1186/s12864-015-2324-4)
Supplement: Additional file 1: — Sequencing information of W. ceti WS08. (DOCX 10 kb) [file 12864_2015_2324_MOESM1_ESM.docx]

| Term / Run | 200 bp fragment | long-mate pair |
| --- | --- | --- |
| Ion Sphere Load | 86% | 90% |
| # of reads | 1,968,915 | 5,857,252 |
| # of sequenced bases | 320,743,478 | 875,722,618 |
| Insert size | N/A | 6 Kkp |
