# Supplementary material for: Comparative genome analysis of Weissella ceti, an emerging pathogen of farm-raised rainbow trout
Source: BMC Genomics. 2015 Dec 22;16:1095. doi: 10.1186/s12864-015-2324-4 (PMC4687380; doi:10.1186/s12864-015-2324-4)
Supplement: Additional file 2: — Assembly information of W. ceti WS08. (DOCX 10 kb) [file 12864_2015_2324_MOESM2_ESM.docx]

| Id | Length | Coverage |
| --- | --- | --- |
| assembly_ws08_c01 | 450,325 | 152.77 |
| assembly_ws08_c02 | 386,182 | 173.32 |
| assembly_ws08_c03 | 114,144 | 156.98 |
| assembly_ws08_c04 | 94,082 | 171.89 |
| assembly_ws08_c05 | 92,185 | 176.85 |
| assembly_ws08_c06 | 53,253 | 151.57 |
| assembly_ws08_c07 | 52,559 | 185.16 |
| assembly_ws08_c08 | 45,792 | 141.31 |
| assembly_ws08_c09 | 31,777 | 179.75 |
| assembly_ws08_c10 | 18,141 | 184.31 |
