# Supplementary figures and images for: Comparative genome analysis of Weissella ceti, an emerging pathogen of farm-raised rainbow trout
Source: BMC Genomics. 2015 Dec 22;16:1095. doi: 10.1186/s12864-015-2324-4 (PMC4687380; doi:10.1186/s12864-015-2324-4)

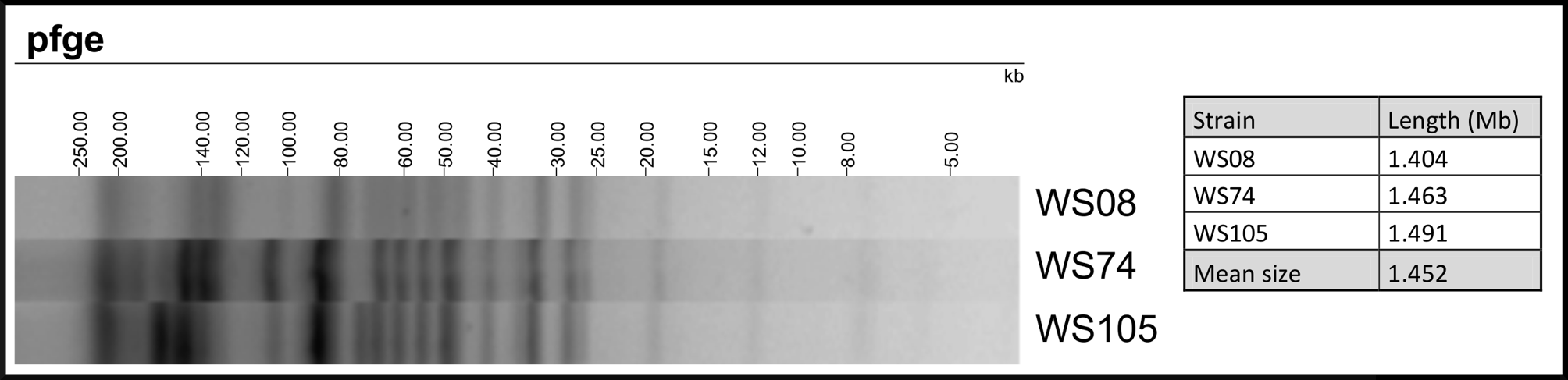

Supplement: Additional file 3: — PFGE and predicted genome sizes of W. ceti WS08, WS74 and WS105 calculated in silico with Bionumerics. (TIFF 220 kb) [file 12864_2015_2324_MOESM3_ESM.tiff]

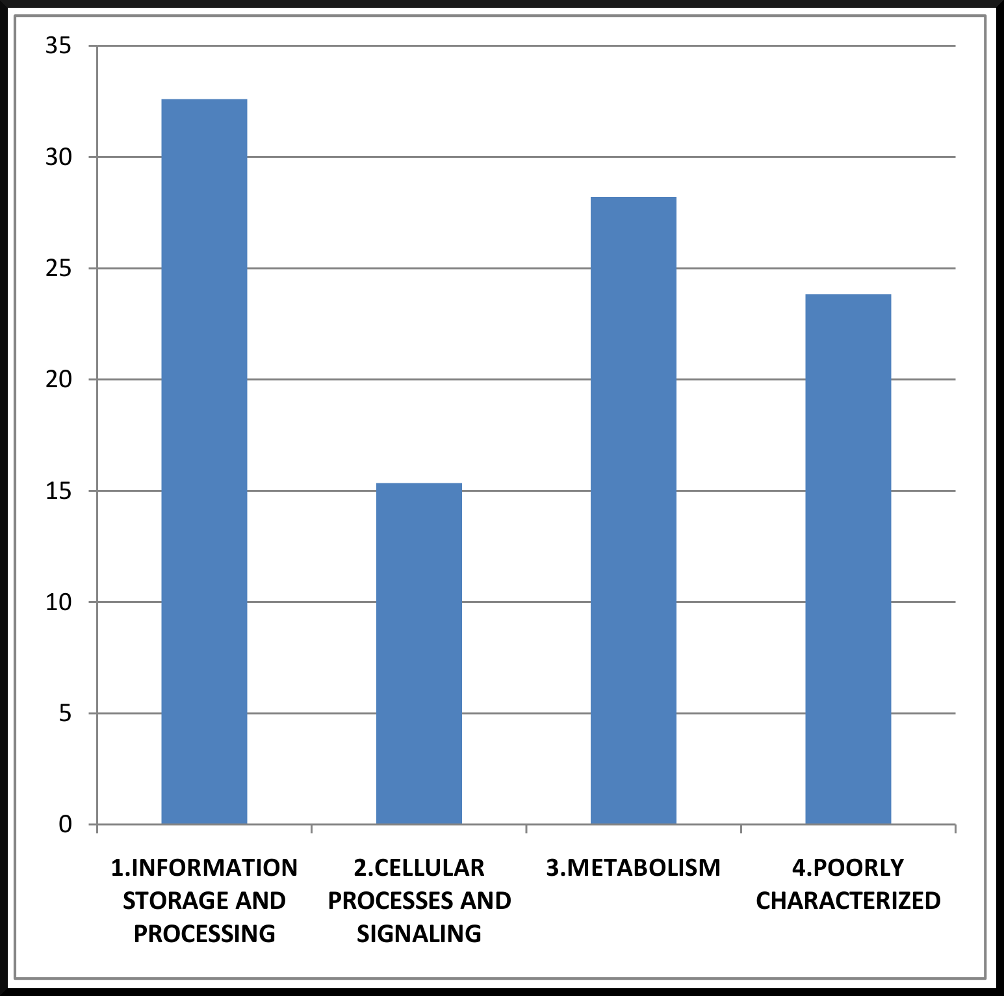

Supplement: Additional file 4: — Core genes of Weissella genus classified by COG functional category. (TIFF 80 kb) [file 12864_2015_2324_MOESM4_ESM.tiff]

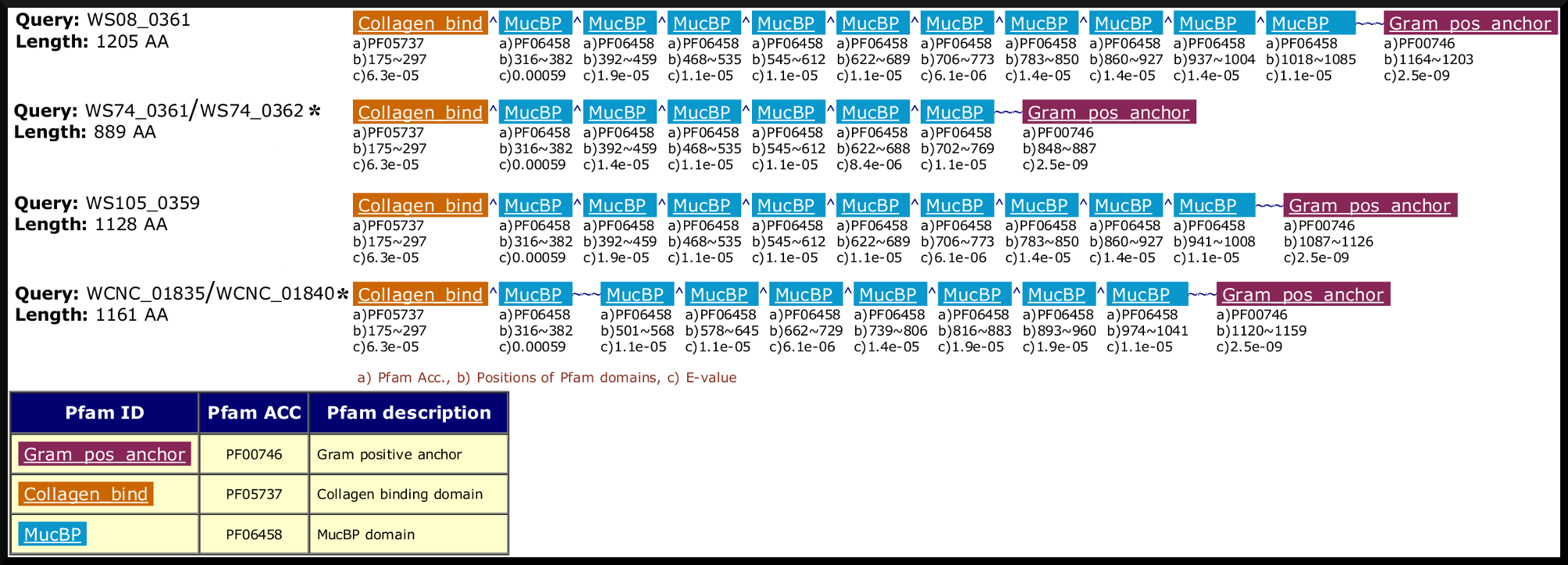

Supplement: Additional file 7: — Visual representation of the MucBP repeated domain in orthologs of WS08_0361 from W. ceti strains. The proteins with “*” were concatenated into one sole sequence before submission to WDAC. (TIFF 512 kb) [file 12864_2015_2324_MOESM7_ESM.tiff]
