# Supplementary material for: Comparative genome analysis of Weissella ceti, an emerging pathogen of farm-raised rainbow trout
Source: BMC Genomics. 2015 Dec 22;16:1095. doi: 10.1186/s12864-015-2324-4 (PMC4687380; doi:10.1186/s12864-015-2324-4)
Supplement: Additional file 8: — Putative multidrug-efflux-related proteins of Weissella ceti. (DOCX 17 kb) [file 12864_2015_2324_MOESM8_ESM.docx]

**Table – Putative multidrug efflux-related proteins from *Weissella ceti***

| WS08 | WS74 | WS105 | NC36 | Prokka annotation | Best blast hit on Uniprot database, ordered by Identity | GEI | Similar to MFS |
| --- | --- | --- | --- | --- | --- | --- | --- |
| WS08_0090 | WS74_0089 | WS105_0090 | WCNC_00817 | MFS family major facilitator transporter | Major facilitator superfamily permease | GEI 1 | Y |
| WS08_0110 | WS74_0109 | WS105_0109 | WCNC_00722 | ABC superfamily ATP binding cassette transporter ATP-binding and permease | ABC transporter ATP-binding protein/permease | - | Y |
| WS08_0169 | WS74_0169 | WS105_0168 | WCNC_00410 | GroEL supressor protein SugE | Cation transporter, small multidrug resistance (SMR) family protein | PAI 1 | Y |
| WS08_0260 | WS74_0260 | WS105_0258 | WCNC_01310 | Hypothetical protein | MATE family multidrug efflux pump protein | - | Y |
| WS08_0394 | WS74_0395 | WS105_0392 | WCNC_01995 | MFS family major facilitator transporter | Multidrug transporter | - | Y |
| WS08_0488 | WS74_0488 | WS105_0486 | WCNC_06017 | Multidrug resistance protein B, MF superfamily | Multidrug resistance protein B | - | Y |
| WS08_0491 | WS74_0492 | WS105_0489 | WCNC_06002 | Major facilitator superfamily permease | Major facilitator superfamily permease | - | Y |
| WS08_0522 | WS74_0523 | WS105_0520 | WCNC_05847 | ABC transporter, ATP-binding protein | Multidrug ABC transporter ATP-binding protein | PAI 2 | Y |
| WS08_0570 | WS74_0571 | WS105_0568 | WCNC_05612 | YusO protein | Transcriptional regulator of fatty acid biosynthesis FabT | PAI 2 | N |
| WS08_0573 | WS74_0574 | WS105_0571 | WCNC_05597 | YxdL protein | Bacteriocin ABC transporter ATP-binding protein | PAI 2 | Y |
| WS08_0592 | WS74_0593 | WS105_0590 | WCNC_05497 | ABC superfamily ATP binding cassette transporter, permease protein | Multidrug ABC transporter permease EcsB | PAI 2 | Y |
| WS08_0593 | WS74_0594 | WS105_0591 | WCNC_05492 | EcsA_3 protein | Multidrug ABC transporter ATP-binding protein EcsA | PAI 2 | Y |
| WS08_0652 | WS74_0654 | WS105_0714 | WCNC_05212 | YxdM protein | Efflux ABC transporter, permease protein | - | Y |
| WS08_0653 | WS74_0655 | WS105_0715 | WCNC_05207 | ABC superfamily ATP binding cassette transporter, ABC protein | Bacitracin ABC transporter ATP-binding protein | - | Y |
| WS08_0746 | WS74_0748 | WS105_0810 | WCNC_04757 | ABC superfamily ATP binding cassette transporter, ABC protein | Putative drug resistance ABC transporter ATP-binding subunit | - | Y |
| WS08_0787 | WS74_0790 | WS105_0851 | WCNC_04557 | Major facilitator superfamily transporter | Major facilitator superfamily protein | - | Y |
| WS08_0966 | WS74_1032 | WS105_1028 | WCNC_02427 | Permease of the major facilitator superfamily protein | Major facilitator superfamily permease | - | Y |
| WS08_1157 | WS74_1226 | WS105_1220 | WCNC_03392 | ABC-type multidrug transport system, ATPase and permease component | Multidrug ABC transporter ATPase/permease LmrD | - | Y |
| WS08_1158 | WS74_1227 | WS105_1221 | WCNC_03397 | ABC superfamily ATP binding cassette transporter, membrane protein | Multidrug ABC transporter ATP-binding protein MsbA | - | Y |
| WS08_1256 | WS74_1327 | WS105_1321 | WCNC_03877 | Lactoylglutathione lyase | Glyoxalase family protein | - | N |
